# Lateral versus supine positioning for proximal femoral nailing of unstable intertrochanteric fractures in geriatric patients: A prospective randomized comparative study

**DOI:** 10.1051/sicotj/2026015

**Published:** 2026-04-29

**Authors:** Wessam Fakhery Ebied, Hesham Ossama Soubih, Yahia Haroun, Karim Atef Salem, Amr Amal Amin, Ahmed Sayed Kotb

**Affiliations:** Orthopedic Surgery Department, Faculty of Medicine, Ain Shams University Cairo Egypt

**Keywords:** Intertrochanteric fracture, Proximal femoral nailing, Lateral decubitus, Traction table, Geriatric fracture

## Abstract

*Background*: Patient positioning for proximal femoral nailing (PFN) in unstable intertrochanteric fractures remains controversial and may influence operative efficiency, radiation exposure, and reduction quality. This study compared lateral decubitus PFN without traction versus the conventional supine traction-table technique in geriatric patients. *Methods*: This prospective randomized comparative study enrolled patients aged >60 years with AO/OTA A2 unstable intertrochanteric fractures who were randomized to supine traction-table PFN (Group A) or lateral decubitus PFN on a radiolucent table (Group B). Primary outcomes were setup time, fluoroscopy (radiation) exposure, and operative time. Secondary outcomes included blood loss, need for open reduction, neck–shaft angle (NSA), tip–apex distance (TAD), and modified Baumgartner reduction quality. *Results*: Setup time was markedly shorter with lateral positioning (13.73 ± 2.26 vs 43.73 ± 6.19 min; *P* < 0.001), and radiation exposure was lower (60.53 ± 15.98 vs 68.48 ± 14.65 s; *P* = 0.023). Blood loss was higher in the lateral group (328.75 ± 84.65 vs 288.75 ± 48.68 mL; *P* = 0.011), and open reduction was more frequent (57.5% vs 17.5%; *P* < 0.001). Operative time was comparable (78.53 ± 15.13 vs 74.48 ± 8.56 min; *P* = 0.145). NSA (135.88 ± 5.94 vs 136.12 ± 6.27°; *P* = 0.864), TAD (23.58 ± 2.14 vs 23.15 ± 1.73 mm; *P* = 0.331), and reduction quality (good: 90% in both; *P* = 1.000) did not differ. *Conclusions*: Lateral decubitus PFN without traction improved setup efficiency and reduced radiation exposure while maintaining comparable radiographic outcomes, at the expense of more frequent open reduction and modestly higher blood loss.

## Introduction

Geriatric intertrochanteric fractures are a major cause of disability and mortality, and their incidence continues to increase with population ageing. Many affected patients have multiple medical comorbidities, which makes prolonged immobilization particularly harmful [1].

Early operative fixation is therefore preferred because it facilitates mobilization and reduces complications related to recumbency, including respiratory and urinary infections, pressure injuries, and venous thromboembolism [2]. In unstable fracture patterns, extramedullary constructs have been associated with higher rates of fixation failure and complications, which has supported the broad adoption of intramedullary devices. The biomechanical advantages of cephalomedullary nails in unstable intertrochanteric fractures have been consistently reported and have driven their use as the implant of choice in many centers [3–6].

Patient positioning during proximal femoral nailing remains debated and may affect reduction quality, fluoroscopy use, and operative efficiency. Supine traction-table positioning provides sustained, controlled traction and is familiar to most operative teams, but it can be associated with traction-related soft-tissue or neurovascular complications and may make access to the entry point and conversion to open reduction more difficult in selected patients, including those with obesity or failed closed reduction [6, 7].

Lateral positioning on a radiolucent table can improve exposure of the entry point, facilitate reaming, and allow easier conversion to open reduction when required, yet it may be more demanding for maintaining reduction and can complicate acquisition of lateral fluoroscopic views [8]. Available comparative evidence has not established the optimal approach, particularly in geriatric patients with unstable intertrochanteric fractures [9, 10].

We hypothesize that proximal femoral nailing performed in the lateral position without a traction table is a safe and efficient alternative to the conventional supine traction-table technique. Hence, this study aimed to compare these two positions in patients over 60 years old with AO/OTA A2 unstable intertrochanteric fractures, focusing on setup time, fluoroscopy time, operative time, and radiographic reduction quality assessed by neck–shaft angle, tip–apex distance, and the modified Baumgartner criteria.

## Patients and methods

### Study design and setting

This prospective, randomized comparative study was conducted at Ain Shams University Hospitals (Cairo, Egypt) between January 2021 and January 2024. The study protocol was approved by the Hospital Research Ethics Committee (Approval code: FMASU R133/2024), and written informed consent was obtained from all participants prior to enrolment.

### Eligibility criteria

Patients aged >60 years presenting with unstable intertrochanteric fractures classified as AO/OTA A2 were eligible for inclusion. Patients were excluded if they had stable intertrochanteric fractures suitable for dynamic hip screw fixation (AO/OTA A1), fractures with subtrochanteric extension, reverse oblique intertrochanteric fractures, associated ipsilateral femoral shaft fractures, pathological fractures, associated ipsilateral hip osteoarthritis, or polytrauma.

### Randomization and allocation concealment

After meeting the eligibility criteria, patients were enrolled and randomly assigned to one of the two treatment groups using Microsoft Excel 16.57 (2021), which implemented simple randomization. Allocation concealment was maintained by an independent research assistant who oversaw group assignment.

### Preoperative assessment

All patients underwent standardized clinical evaluation, including history taking and physical examination. Radiographic evaluation included hip, pelvis, and full-length femoral radiographs to confirm fracture characteristics and guide operative planning.

### Operative technique and interventions

All procedures were performed under anaesthesia. In both groups, fracture fixation was achieved using a short proximal femoral nail (180 mm) from the same manufacturer. The implant configuration included a lag screw and an anti-rotation screw for head–neck fixation, as well as two distal locking screws inserted using a targeting device.

**Figure 1:**
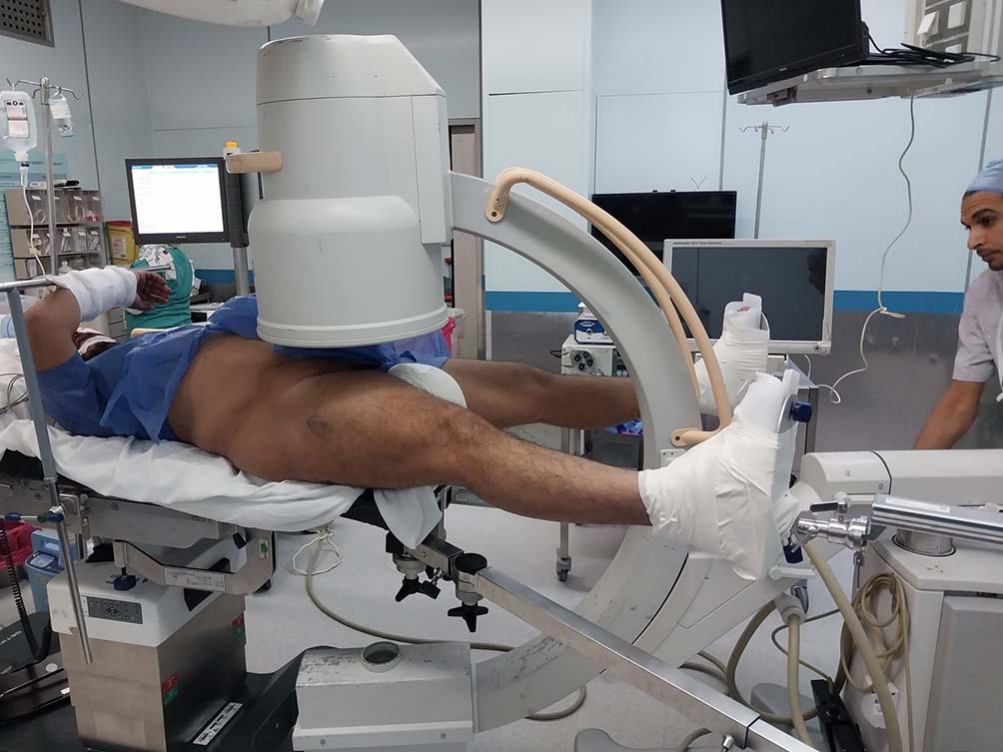
Supine decubitus patient positioning in a traction table with C-arm alignment for AP hip fluoroscopic imaging.

**Figure 2:**
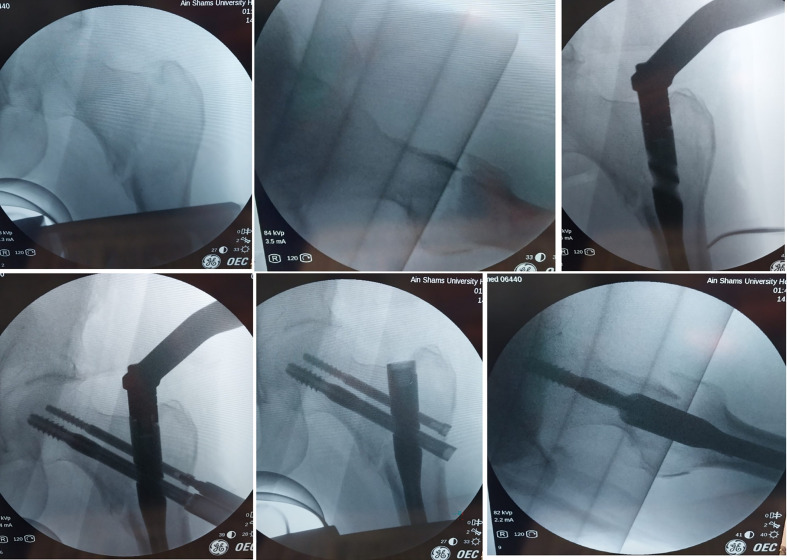
Fluoroscopic images of closed reduction and nail insertion in a patient of the supine position group.

In Group A (supine traction-table group), patients were positioned supine on a radiolucent traction table. The fractured limb was secured in a fracture boot, and the contralateral limb was placed in a lithotomy position or put in a fracture boot (Figure 1). Closed reduction was performed using longitudinal traction and internal rotation under image intensifier guidance. Trunk adduction was used to facilitate access to the entry point (Figure 2).

**Figure 3:**
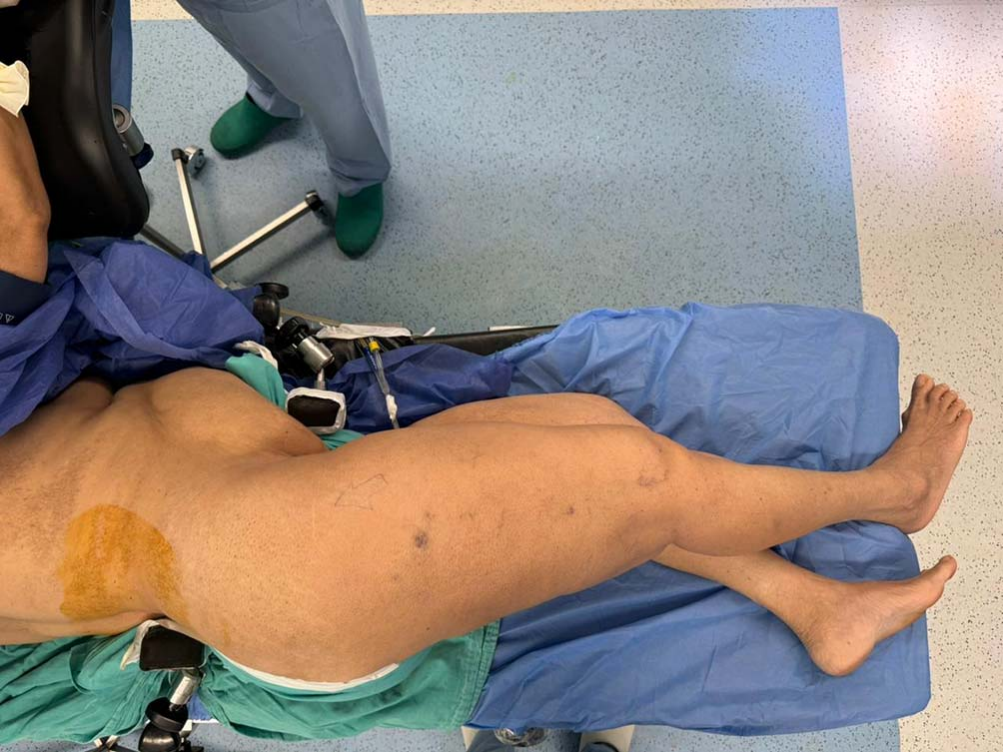
Pelvic stabilization was achieved using anterior and posterior bolsters in the lateral position group.

**Figure 4:**
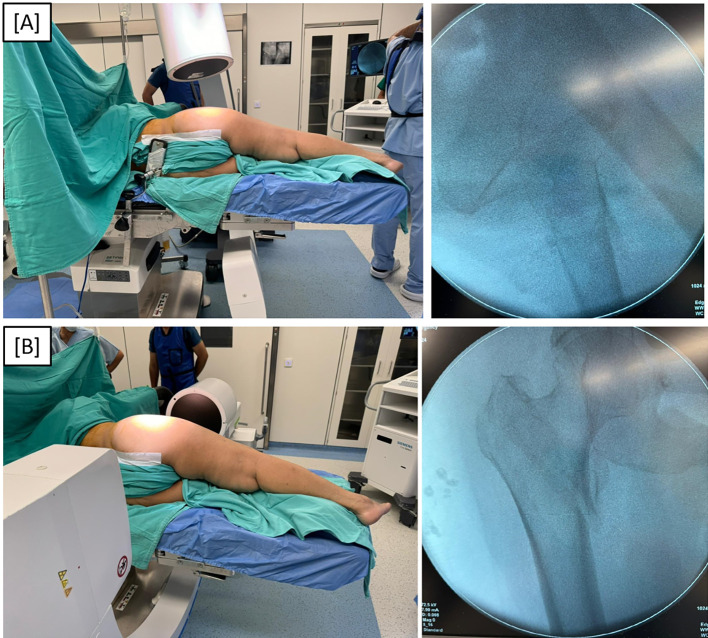
(A) Lateral fluoroscopic imaging in the lateral position group. (B) Anteroposterior fluoroscopic imaging in the lateral position group.

**Figure 5:**
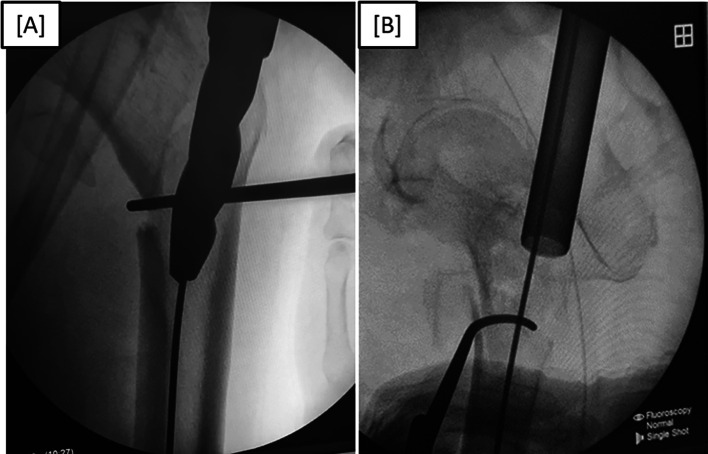
(A) Anteroposterior fluoroscopic view of left intertrochanteric fracture reduction using a bone hook during proximal femoral nail insertion. (B) Lateral fluoroscopic view of left intertrochanteric fracture reduction using a bone hook during proximal femoral nail insertion.

In Group B (lateral position group), patients were positioned in the lateral decubitus position on a radiolucent table with the fractured limb uppermost. The contralateral limb was positioned down and flexed at the hip and knee to clear the field in the lateral x-ray view. Pelvic stabilization was achieved using anterior and posterior bolsters in a manner similar to positioning for hip arthroplasty (Figure 3), with adequate padding of bony prominences. Lateral imaging of the hip requires tilting the C-arm 30° cephalad to get a longer neck profile, with the beam source under the table to facilitate the C-arm movement. The normal hip will appear flexed and larger in size due to proximity to the beam source. The femoral head Reduction was attempted using gentle longitudinal traction under fluoroscopy. Reduction is confirmed in both AP and lateral fluoroscopy views (Figures 4A, 4B). When acceptable closed reduction could not be achieved, open reduction was performed through a limited lateral approach, and a hook was used to reduce the medialized femoral neck relative to the shaft under fluoroscopic guidance (Figures 5A, 5B) before proceeding with nail insertion.

### Outcomes and definitions

The study compared setup time, fluoroscopy time, operative time, and radiographic reduction quality. Reduction quality was evaluated using the neck–shaft angle (NSA), tip–apex distance (TAD), and the modified Baumgartner criteria.

Setup time was defined as the time required for positioning the patient, preparation, and draping; in the traction-table group, this interval also included fracture reduction performed under image intensifier during positioning. Operative time was defined as the time from skin incision to completion of skin closure. Fluoroscopy time was defined as the total duration of image intensifier use during both setup and operative periods.

### Statistical methods

Data were analyzed using IBM SPSS Statistics (IBM Corporation, Somers, New York, USA). Quantitative data were summarized as mean ± standard deviation for parametric variables and as median with interquartile range for non-parametric variables. Categorical variables were summarized as numbers and percentages. Comparisons between groups were performed using the chi-square test and/or Fisher’s exact test for categorical variables. For continuous parametric variables, the independent *t*-test was used. A two-sided *P* value <0.05 was considered statistically significant.

## Results

**Figure 6:**
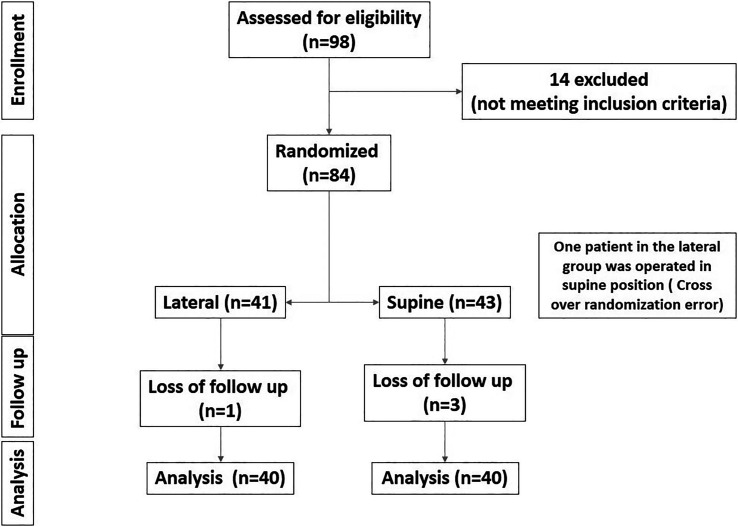
Consort flow diagram of the enrolled patients.

In this study, 98 patients were assessed for eligibility, and 14 were excluded for not meeting the inclusion criteria. The remaining 84 patients were randomized equally to the lateral and supine groups (42 patients each). During follow-up, one patient in the lateral group and three patients in the supine group were lost to follow-up. In our study, “lost to follow up” means proper post-operative radiographs could not be obtained to extract the study’s primary radiological outcomes. A patient originally randomized to the lateral group was operated in the supine group due to a crossover error. This crossover patient was included in the supine group as we used Per-Protocol analysis. Consequently, 40 patients in each group were included in the final analysis (Figure 6).

Baseline characteristics were comparable between the lateral and supine groups, with no significant differences in age (*P* = 0.374), sex (*P* = 0.572), pre-operative comorbidities (*P* = 0.745), thyroidectomy history (*P* = 1), smoking status (*P* = 1), pulmonary embolism (*P* = 0.556), ischemic heart disease (*P* = 1), cardiac comorbidity (*P* = 0.576), hepatitis C virus infection (*P* = 0.762), diabetes mellitus (*P* = 0.644), hypertension (*P* = 0.799), ASA class (*P* = 0.602), or AO fracture class distribution (*P* = 1) Table 1.

**Table 1:** Baseline demographic and clinical characteristics and fracture classification between the studied groups.

		Lateral (*n* = 40)	Supine (*n* = 40)	*P*-value
Age	Mean ± SD	74.63 ± 6.33	73.38 ± 6.17	0.374
Gender				
Female	*n* (%)	24 (60.0)	22 (55.0)	0.572
Male	*n* (%)	16 (40.0)	18 (45.0)	
Pre-operative comorbidities	*n* (%)	35 (87.5)	34 (85)	0.745
Thyroidectomy	*n* (%)	2 (5.7)	2 (5.9)	1
Smoker	*n* (%)	3 (8.6)	3 (8.8)	1
PE	*n* (%)	2 (5.7)	1 (2.9)	0.556
ISHD	*n* (%)	7 (20)	7 (20.6)	1
Cardiac	*n* (%)	9 (25.7)	7 (20.6)	0.576
HCV	*n* (%)	7 (20)	6 (17.6)	0.762
Diabetes mellitus	*n* (%)	26 (74.3)	24 (70.6)	0.644
Hypertension	*n* (%)	30 (85.7)	29 (85.3)	0.799
ASA	Mean ± SD	2.65 ± 0.86	2.55 ± 0.85	0.602
AO class				
31A1.2	*n* (%)	7 (17.5)	8 (20.0)	1
31A1.3	*n* (%)	8 (20.0)	8 (20.0)	
31A2.1	*n* (%)	4 (10.0)	3 (7.5)	
31A2.2	*n* (%)	11 (27.5)	10 (25.0)	
31A2.3	*n* (%)	3 (7.5)	3 (7.5)	
31A3.1	*n* (%)	2 (5.0)	3 (7.5)	
31A3.2	*n* (%)	2 (5.0)	2 (5.0)	
31A3.3	*n* (%)	3 (7.5)	3 (7.5)	

*n*: number, SD: standard deviation, PE: pulmonary embolism, ISHD: ischemic heart disease, HCV: hepatitis C virus, ASA: American Society of Anesthesiologists.

Setup time was significantly shorter in the lateral group than in the supine traction-table group (13.73 ± 2.26 vs 43.73 ± 6.19 min, *P* < 0.001). Blood loss was significantly higher in the lateral group (328.75 ± 84.65 vs 288.75 ± 48.68 mL, *P* = 0.011). Radiation exposure was significantly lower in the lateral group (60.53 ± 15.98 vs 68.48 ± 14.65 s, *P* = 0.023). The need for open reduction was significantly more frequent in the lateral group (57.5% vs 17.5%, *P* < 0.001). Surgical time did not differ significantly between groups (*P* = 0.145) Table 2.

**Table 2:** Operative and intraoperative outcomes between the studied groups.

		Lateral (*n* = 40)	Supine (*n* = 40)	*P*-value
Set up time (minutes)	Mean ± SD	13.73 ± 2.26	43.73 ± 6.19	<0.001*
Surgical time (minutes)	Mean ± SD	78.53 ± 15.13	74.48 ± 8.56	0.145
Blood Loss (mL)	Mean ± SD	328.75 ± 84.65	288.75 ± 48.68	0.011*
Radiation exposure (seconds)	Mean ± SD	60.53 ± 15.98	68.48 ± 14.65	0.023*
Need for open reduction	*n* (%)	23 (57.5)	7 (17.5)	<0.001*

*n*: number, SD: standard deviation, *: Significant *P*-value.

No significant differences were observed between groups in neck–shaft angle (*P* = 0.864), radiographic reduction quality (*P* = 1.000), or tip–apex distance (*P* = 0.331) Table 3.

**Table 3:** Radiographic reduction quality and implant positioning parameters between the studied groups.

		Lateral (*n* = 40)	Supine (*n* = 40)	*P*-value
NSA (deg)	Mean ± SD	135.88 ± 5.94	136.12 ± 6.27	0.864
Reduction quality				
Acceptable	*n* (%)	4 (10.0)	4 (10.0)	1
Good	*n* (%)	36 (90.0)	36 (90.0)	
TAD	Mean ± SD	23.58 ± 2.14	23.15 ± 1.73	0.331

*n*: number, SD: standard deviation, NSA: neck–shaft angle, deg: degrees, TAD: tip–apex distance.

## Discussion

Intertrochanteric fractures in the geriatric population are a major cause of morbidity and mortality. Their incidence is expected to rise with increasing life expectancy [1, 2]. Management is often complex and typically requires a multidisciplinary approach, including orthogeriatric input. The primary treatment goal is stable surgical fixation that permits early mobilization, and intramedullary nailing is increasingly used for this purpose. Patient positioning for femoral intramedullary nailing is commonly either supine on a traction table or lateral on a radiolucent table. The supine traction-table position remains the conventional approach [5, 6].

Supine positioning on a traction table offers controlled limb traction, may facilitate management of associated injuries, and can allow the procedure to be performed with minimal assistance. However, traction-table use carries recognized risks, including soft-tissue and perineal injury, neurovascular complications (notably pudendal and common peroneal nerve injuries), and compartment syndrome. Access to the proximal femoral entry point may also be more difficult, particularly in obese patients, and conversion to open reduction can be technically challenging in the supine position. By contrast, lateral positioning without a traction table can improve access to the entry point – especially in obese patients – and can facilitate conversion to open reduction when required. This approach, however, may be more demanding for anesthesia and fluoroscopy acquisition [11, 12].

Evidence supporting lateral positioning for intramedullary nailing remains limited, and direct comparisons between lateral decubitus and supine traction-table positioning in the treatment of unstable intertrochanteric fractures are relatively few [2, 8, 9].

In this study, we compared proximal femoral nailing for unstable intertrochanteric fractures in patients aged over 60 years performed in the supine position on a traction table versus the lateral decubitus position on a radiolucent table without traction.

We found that radiographic outcomes were comparable between groups, with no significant differences in neck–shaft angle, tip–apex distance, or overall reduction quality. Operative workflow favored the lateral technique, which achieved a substantially shorter setup time, while operative time was similar between positions (Table 3). Hook-assisted mini-open reduction was required more frequently in the lateral group, which likely explains the slightly higher blood loss that was statistically significant but not clinically meaningful. This is due to failure to maintain closed reduction due to the absence of traction. Radiation exposure was higher in the supine traction-table group.

These findings align with several published comparisons. Sönmez et al. [2] evaluated 82 patients (mean age 78 years) treated with intramedullary nailing and reported shorter operative time in the lateral group than the supine group (28.70 ± 7.11 vs 32.08 ± 7.33), with shorter setup time in the lateral decubitus group (17.65 min) than the traction-table group (21.67 min). They found no significant between-group differences in TAD or radiographic reduction quality. Xue et al. [9], in a study of 120 intertrochanteric fractures treated with proximal femoral nailing, reported significantly lower blood loss, shorter operative time, and shorter fluoroscopy time in the lateral group. Souza et al. [10] retrospectively assessed radiographic reduction and cephalic implant position after proximal femoral nailing and found unacceptable NSA values more frequently in the traction-table group (11 patients, 61.1%) than in the lateral group (one patient, 5.3%), while TAD and cephalic component position were similar.

Additional retrospective data support potential efficiency advantages of lateral positioning. Kuru [13] compared lateral versus supine positioning for PFN in patients older than 65 years and found significantly shorter operative time and fluoroscopy time in the lateral group, with no significant difference in postoperative bleeding. Güzel et al. [14] retrospectively studied 120 patients (65–90 years) with unstable intertrochanteric fractures treated with PFN and reported lower blood loss, fewer intraoperative fluoroscopy images, and a shorter incision in the lateral-position group.

Finally, this study had some limitations. It was a single-center study, and procedures were performed by multiple surgeons and radiographers with varying levels of experience, which may have influenced operative workflow and fluoroscopy use. The second limitation is the lack of data regarding Body Mass Index (BMI). Given that obesity can complicate surgical exposure and influence mechanical load on internal fixation, the absence of this metric prevents a granular analysis of how body habitus may have affected our specific fracture management outcomes. In addition, the follow-up was insufficient to evaluate key outcomes such as complications, union rates, and functional recovery (e.g., Harris Hip Score). Therefore, given the limited comparative literature, larger prospective multicenter studies with standardized protocols are needed, and variability across studies in operative time, blood loss, open reduction rates, and radiation exposure may reflect differences in fracture morphology, patient BMI, and team expertise.

## Conclusions

Lateral decubitus positioning without traction for proximal femoral nailing in geriatric patients with unstable intertrochanteric fractures appears to be a safe and efficient alternative to supine traction-table fixation. However, it is technically demanding and depends on the surgeon's experience, coordinated team performance, and skilled fluoroscopic imaging.

## Data Availability

Data are available from the corresponding author upon reasonable request.
